# Intensity modulated radiotherapy induces pro-inflammatory and pro-survival responses in prostate cancer patients

**DOI:** 10.3892/ijo.2014.2260

**Published:** 2014-01-17

**Authors:** HOUSSEIN EL-SAGHIRE, CHARLOT VANDEVOORDE, PIET OST, PIETER MONSIEURS, ARLETTE MICHAUX, GERT DE MEERLEER, SARAH BAATOUT, HUBERT THIERENS

**Affiliations:** 1 Radiobiology Unit, Molecular and Cellular Biology, Belgian Nuclear Research Centre (SCK·CEN), Mol;; 2 Departments of Basic Medical Sciences, Ghent University, Gent, Belgium; 3 Molecular Biotechnology, Ghent University, Gent, Belgium; 4 Department of Radiation Oncology, Ghent University Hospital, Gent, Belgium

**Keywords:** IMRT, low dose, ionizing radiation, inflammatory response, prostate cancer, radiotherapy, microarray, γ-H2AX

## Abstract

Intensity modulated radiotherapy (IMRT) is one of the modern conformal radiotherapies that is widely used within the context of cancer patient treatment. It uses multiple radiation beams targeted to the tumor, however, large volumes of the body receive low doses of irradiation. Using γ-H2AX and global genome expression analysis, we studied the biological responses induced by low doses of ionizing radiation in prostate cancer patients following IMRT. By means of different bioinformatics analyses, we report that IMRT induced an inflammatory response via the induction of viral, adaptive, and innate immune signaling. In response to growth factors and immune-stimulatory signaling, positive regulation in the progression of cell cycle and DNA replication were induced. This denotes pro-inflammatory and pro-survival responses. Furthermore, double strand DNA breaks were induced in every patient 30 min after the treatment and remaining DNA repair and damage signaling continued after 18–24 h. Nine genes belonging to inflammatory responses (*TLR3, SH2D1A* and *IL18*), cell cycle progression (*ORC4, SMC2* and *CCDC99*) and DNA damage and repair (*RAD17, SMC6* and *MRE11A*) were confirmed by quantitative RT-PCR. This study emphasizes that the risk assessment of health effects from the out-of-field low doses during IMRT should be of concern, as these may increase the risk of secondary cancers and/or systemic inflammation.

## Introduction

Currently, intensity modulated radiotherapy (IMRT) is a widely applied conformal radiotherapy modality; in contrast to conventional radiotherapy, IMRT uses multiple beams across the target field of treatment. This reduces the volume of tissues receiving high doses, but a greater volume of normal tissues still receives low doses of radiation (1–3). It is estimated that IMRT can contribute to 1.5% increased risk of secondary cancers by 10 years following treatment (4). However, these figures were considered to be overestimated because the calculations of these risks were based on the long-term data obtained from the follow-up of atomic bomb survivors. This population was exposed to a single whole body dose, while IMRT patients receive fractionated doses to specific body parts (5). Besides, other studies moderated the therapeutic effect of IMRT over its potential health side effects (6,7).

Microarrays and DNA damage studies, through measuring the Ser 139 phosphorylated form of histone H2AX (γ-H2AX), are emerging applications in the field of radiation biology and biodosimetry. Gene expression studies improved the knowledge on cellular responses to both high and low radiation doses (8–11). On the other hand, γ-H2AX foci immunodetection has been described as useful quantitative biomarker of human low-level radiation exposure (12).

In this study, we address the question of understanding the whole blood tissue biological responses in prostate cancer patients receiving low doses of ionizing radiation during IMRT. It is the first study that combines DNA damage and microarray investigations on whole blood samples collected *in vivo* from patients receiving low doses over a large part of the body. It highlights the mechanisms and the possible health effects involved in response to low doses of ionizing radiation. For the DNA damage assessment γ-H2AX foci were scored. For the analysis of the microarray data, we applied a holistic approach, namely Gene Set Enrichment Analysis (GSEA) that it is known to overcome many of the limitations in individual gene pathway analysis, discussed thoroughly by Subramanian and colleagues (13). In addition, we used differentially expressed genes for Exploratory Gene Association Networks (EGAN) analysis.

## Materials and methods

### Patients and sample collection

The study population consisted of 8 prostate cancer patients treated with step and shoot-IMRT (ss-IMRT) (Elekta Synergy linear accelerator) at the Department of Radiation Oncology (Ghent University Hospital, Belgium) between March and May 2013. A dose per fraction to the tumor was 2.09 Gy. After obtaining written approval of the ethics committee at Ghent University Hospital and signed informed consent, blood samples were taken at different time-points. Blood sampling for the γ-H2AX foci was performed in heparin vacutainer tubes before and 30 min after the first fraction, blood sampling for the whole genome expression analysis was performed in EDTA vacutainer tubes before the first and second fraction, 18–24 h after the first fraction.

### Dose calculation

The equivalent total body blood dose (DETB) was calculated for each patient based on the treatment planning data. To this end, the mean dose within the skin contour of the scanned volume was normalised to the patient mass. As liver, heart/large blood vessels and lungs contain together 38.5% of the total blood volume it was assumed that 61.5% of the blood pool is distributed uniformly over the rest of the body.

### γ-H2AX scoring

The procedure for the γ-H2AX foci assay on T-lymphocytes is described in detail in a previous report (14). Foci analysis was performed with the Cytovision v.2.8 Software 2002 (Applied Imaging, USA) and an Olympus BX60 fluorescent microscope was used with a 100x/1.30 oil lens. Several images of one slide were captured with a digital camera (Applied Imaging), 10 Z-stacks with 1.03 *μ*m spacing was used.

### RNA isolation for microarray gene expression studies

Collected venous blood (4-ml/time-point) was passed through a LeukoLOCK™ filter (Life Technologies, USA), washed with PBS and leukocytes were stabilized with RNAlater^®^. Filters were capped and stored at −20°C. RNA was isolated using LeukoLOCK™ Isolation System (Life Technologies) according to the manufacturer’s instructions. RNA was quantified using a Nanodrop 2000 (Thermo Scientific, USA) spectrophotometer, the quality was assessed with Agilent 2100 Bioanalyzer. All RNA samples had ≥8.5 as an integrity number.

### Microarray assay

Using the Ambion^®^ WT Expression kit (Ambion, USA), cDNA was prepared from 10 *μ*g of purified cRNA, originally synthesized and purified from 0.25 *μ*g of total RNA, following the manufacturer’s instructions. The cDNA (2.75 *μ*g) was used for fragmentation and labeling using GeneChip^®^ Terminal Labeling kit (Affymetrix, USA). Using GeneChip^®^ Hybridization, Wash and Stain kit (hybridization module) (Affymetrix), and hybridization controls (Affymetrix), fragmented and labeled cDNA was hybridized to Human Gene 1.0 ST arrays (Affymetrix). After hybridization with rotation for 16 h at 45°C, arrays were washed and stained, according to the manufacturer’s instructions, using GeneChip^®^ Hybridization, Wash and Stain kit (stain module) (Affymetrix). Finally, arrays were scanned immediately using Affymetrix GeneChip^®^ Scanner.

### Microarray data processing

Raw Affymetrix data were preprocessed using Partek Genomics Suite v6.6 (Partek Inc., USA). Briefly, Robust Multichip Average (RMA) was used for background correction followed by quantile normalization and summarization of multiple probe intensities for each probeset using the median polish approach (15). Gene expression values were obtained by the one-step Tukey method.

### Functional analysis - GSEA

GSEA calculates an enrichment score (ES) reflecting the overrepresentation of a certain gene set at the top or bottom of a ranked list of genes found in the expression dataset of two classes. This method applies the Kolmogorov-Smirnov test to find deviation between two distributions. Information on GSEA was reported previously (13). Briefly, genes are ranked using signal-to-noise ratio. Using Kolmogorov-Smirnov statistics, pre-defined sets of genes are scored and significance is tested by empirical permutation followed by correction for multiple hypotheses. The Reactome database was used as reference background for the implemented analysis. In total, the data were analyzed against 674 gene sets downloaded from the Molecular Signature Database (MSigDB) (http://www.broadinstitute.org/gsea/msigdb/index.jsp). The GSEA software parameters were set to their default values. The statistical significance of the normalized enrichment score (NES) associated to each gene set was assessed through 1,000 random permutations of the phenotypic labels. FDR (false discovery rate) value <0.05 was used as a cut-off value for assessing the statistical significance of the estimates. For gene set networks, we used the Enrichment Map plug-in (16) for Cytoscape Desktop program (http://baderlab.org/Software/EnrichmentMap/). Gene sets with FDR values <0.05 and having ≥50% overlapping genes are represented in the network.

### Functional analysis - Exploratory Gene Association Networks

To test for differential expression between different irradiated conditions and reference conditions (no irradiation) we used repeated measures ANOVA. Differentially expressed genes were defined with a p-value cutoff with a false discovery rate of <0.05. Differentially expressed genes were analyzed using Exploratory Gene Association Networks (EGAN, The Regents of the University of California) software to determine differentially regulated pathways. P-values were corrected using Westfall-Young minP method. P-values <0.05 were considered significant. For clearer illustrations, not all genes belonging to each pathway are shown in the figures.

### Quantitative RT-PCR validation

For quantitative real-time (RT-PCR) confirmation, we selected nine different genes that were shown to be differentially expressed and contributed to the pathway enrichment of immune signaling, DNA damage and repair and cell cycle progression. Briefly, cDNA was prepared from 0.25 *μ*g of total RNA using Ambion^®^ WT Expression kit (Ambion) following the manufacturer’s instructions. RT-PCR was performed using TaqMan^®^ Gene Expression assays (Applied Biosystems, USA). Each TaqMan assay was run in duplicate for each diluted cDNA sample using TaqMan^®^ Fast Advanced Master Mix (Applied Biosystems). The reactions were run on ABI 7500 Fast RT-PCR system following the manufacturer’s recommended PCR program: 95°C for 20 sec, followed by 40 cycles of 95°C for 3 sec and 60°C for 30 sec. Relative expression values were calculated by Pfaffl (17) method normalized to *PGK1* levels. Relative expression levels were tested for statistical significance using paired t-test, genes having p-values <0.05 were considered significant.

## Results

**Figure 1.**
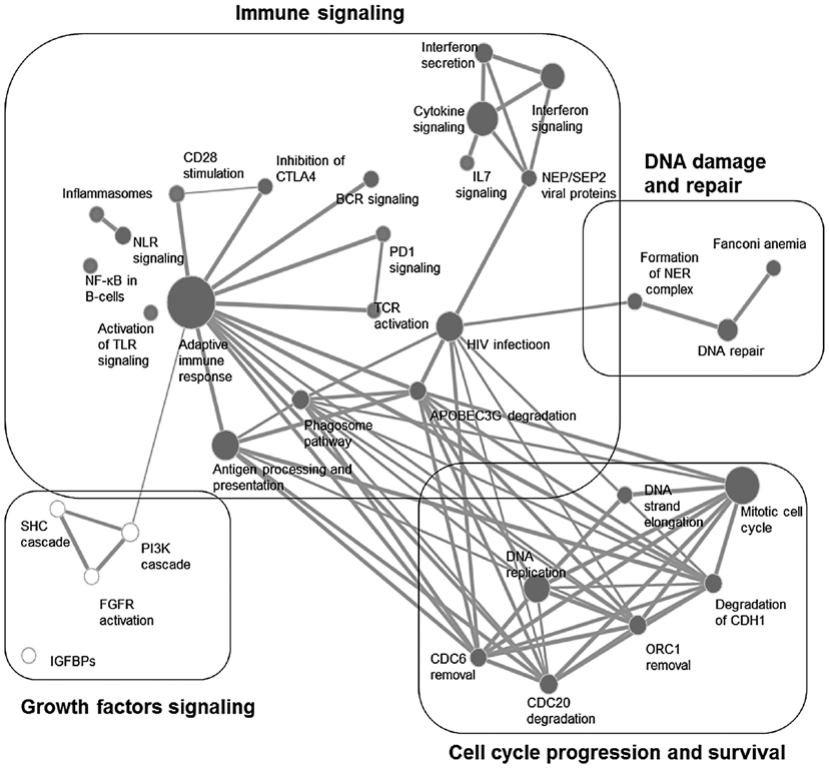
Enrichment map analysis of GSEA results. Each gene set is represented by a node with different size, proportional to the number of genes; the connecting line represents the percentage of overlap and its thickness represents the percentage of overlapping. Black nodes represent upregulated gene sets, whilst white nodes represent downregulated gene sets. A combination of two cut-offs was applied: 5% FDR and a minimum of 50% gene overlapping.

**Figure 2.**
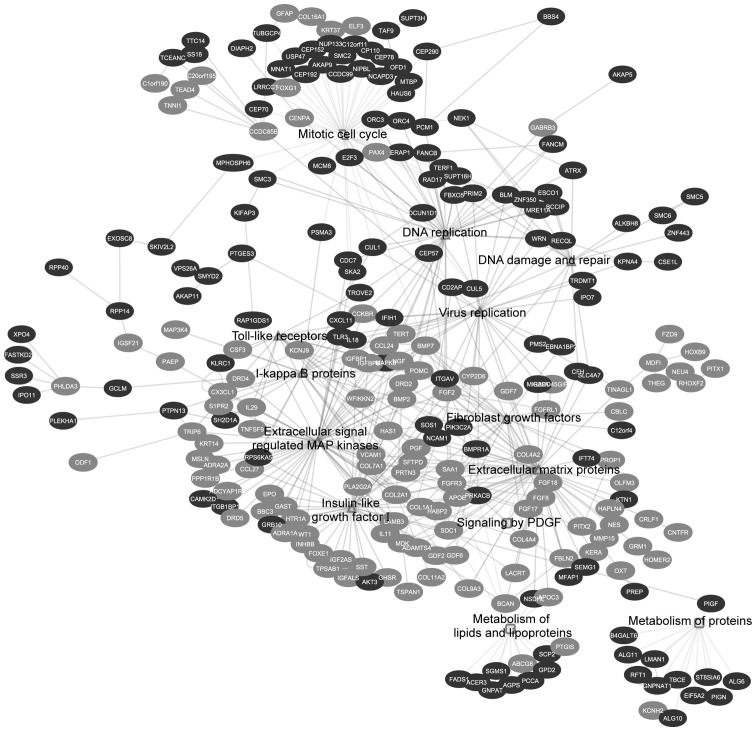
EGAN analysis showing all the differentially expressed enriched pathway genes. Each circle represents a gene. Dark gray circles are upregulated genes; light gray circles are downregulated genes. The lines represent connections between different genes belonging to different pathways.

Based on the treatment planning data the equivalent total body dose of one fraction amounted to 30.97±8.12 mGy (Table III). GSEA enrichment map analysis showed interconnections of 4 different signal transduction categories; these are immune signaling, growth factors signaling, cell cycle progression and survival, as well as DNA damage and repair (Fig. 1). On the other hand, EGAN analysis showed the biological response is divided into three different categories: growth factors and cell cycle progression, viral and immune signaling and metabolism (Table II and Fig. 2).

### Low doses of ionizing radiation induces pro-inflammatory response via the activation of viral, adaptive and innate immune signaling

**Figure 3.**
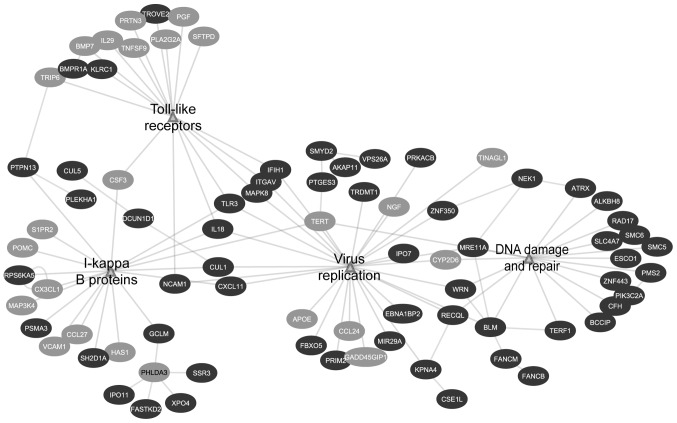
EGAN analysis showing the viral response network. The viral response network is composed from virus replication, IκB proteins, toll-like receptors and DNA damage and repair pathways. Each circle represents a gene. Dark gray circles are upregulated genes; light gray circles are downregulated genes. The lines represent connections between different genes belonging to different pathways.

HIV infection and gene sets belonging to the adaptive immune response contributed mainly to the enrichment of the immune signaling cluster (Table I). The involvement of the viral infection response, along with interferon signaling and secretion and APOBEC3G degradation denotes the induction of an inflammatory response accompanied by DNA damage; the HIV infection node shared a common edge with the DNA damage and repair gene sets (Fig. 1). Furthermore, the enrichment map analysis showed involvement of adaptive immune response activation, particularly CD28 stimulation that works in an opposite way with CTLA4, leading to T-cell receptor activation and cytokine secretion (Table I and Fig. 1). In addition, innate immune gene sets were significantly modulated; these include phagosome pathway, inflammasomes, toll-like receptors and NOD-like receptors (Table I and Fig. 1). Similar to GSEA, EGAN analysis showed the enrichment of signaling involved in viral immune responses. The viral signaling network was composed of several immune-related pathways, namely virus replication, IκB proteins and toll-like receptors. Furthermore, it showed connection with DNA damage and repair node, which is a characteristic of a viral response (Fig. 3 and Table II).

Among the upregulated genes that contributed to the positive regulation of inflammatory response are *SH2D1A, TLR3* and *IL18* (Figs. 3 and 7A).

### Low doses of ionizing radiation induces pro-survival response via immune-stimulation and cell cycle progression responses downstream growth factors signaling

**Figure 4.**
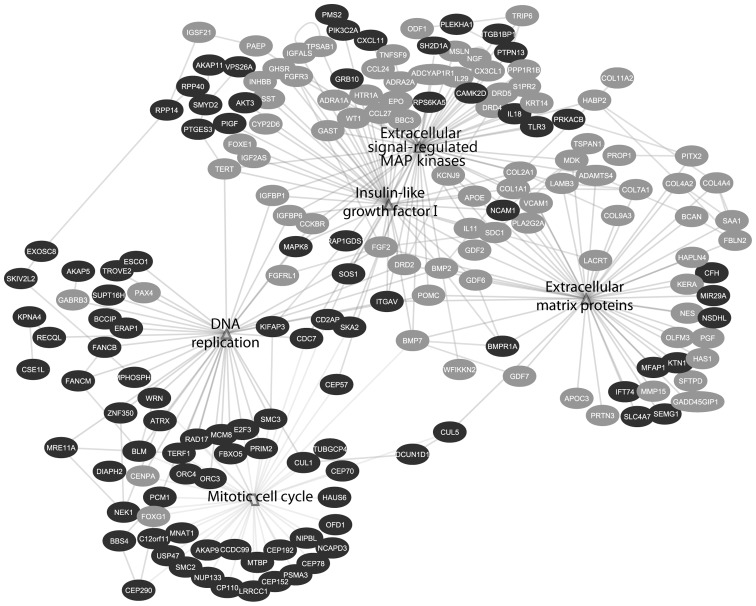
EGAN analysis showing the network between growth factor signaling (fibroblast growth factors, insulin growth factor I, extracellular matrix proteins and ERK MAPKs) and their effect on the positive regulation of cell cycle (mitotic cell cycle and DNA replication). Each circle represents a gene. Dark gray circles are upregulated genes; light gray circles are downregulated genes. The lines represent connections between different genes belonging to different pathways.

Individual gene pathways analysis showed the downregulation of several growth factors like fibroblast growth factors (FGFs), insulin growth factor I (IGF-I), extracellular proteins and platelet-derived growth factor signaling (PDGF) (Table II). Downstream to growth factor signaling, DNA replication and mitotic cell cycle networks were shown to be upregulated (Fig. 4).

**Figure 5.**
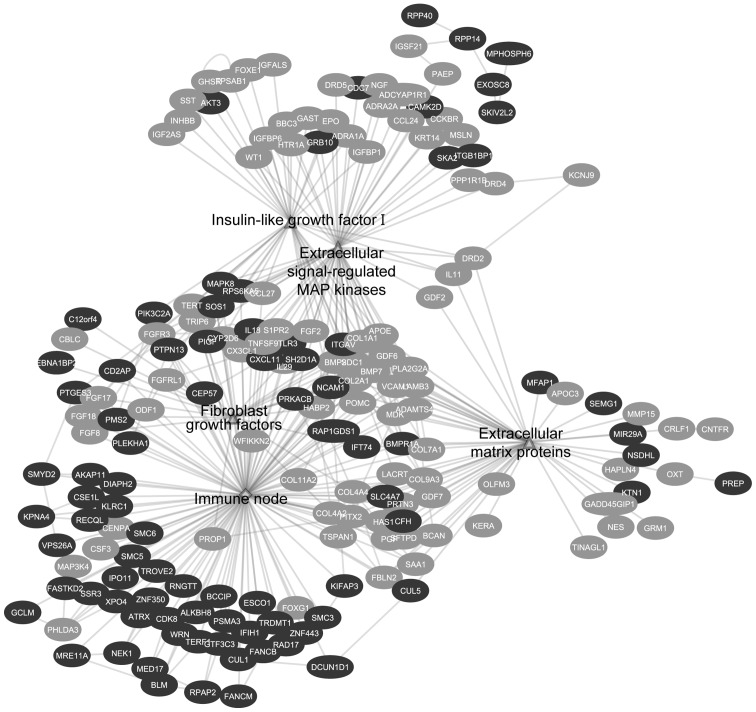
EGAN analysis showing the network between growth factors and their effect on immune-stimulation. The ‘Immune node’ is the viral response network from Fig. 3. Each circle represents a gene. Dark gray circles are upregulated genes; light gray circles are downregulated genes. The lines represent connections between different genes belonging to different pathways.

**Figure 6.**
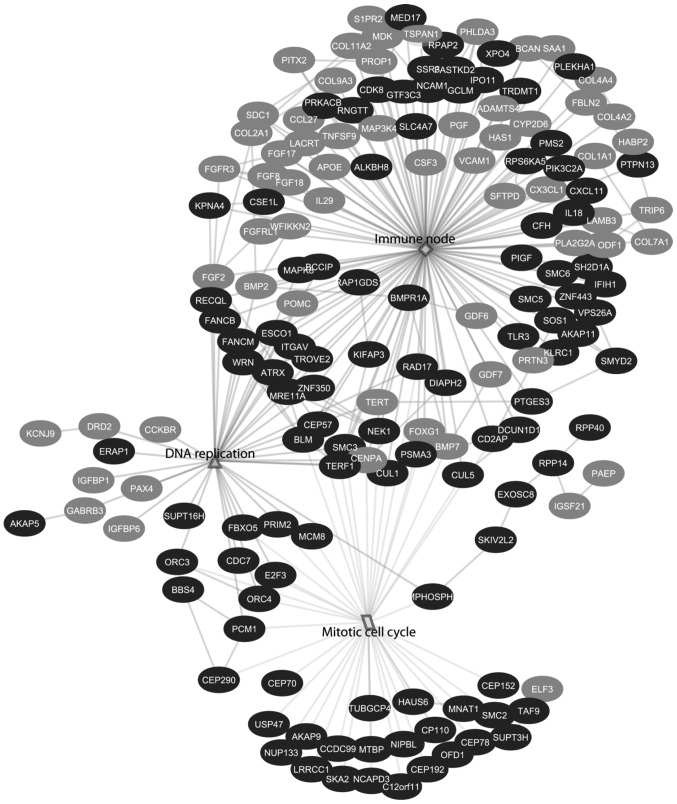
EGAN analysis showing the network between immune-stimulation and their effect on cell cycle progression (mitotic cell cycle and DNA replication). The immune node is the viral response network from Fig. 3. Each circle represents a gene. Dark gray circles are upregulated genes; light gray circles are downregulated genes. The lines represent connections between different genes belonging to different pathways.

**Figure 7.**
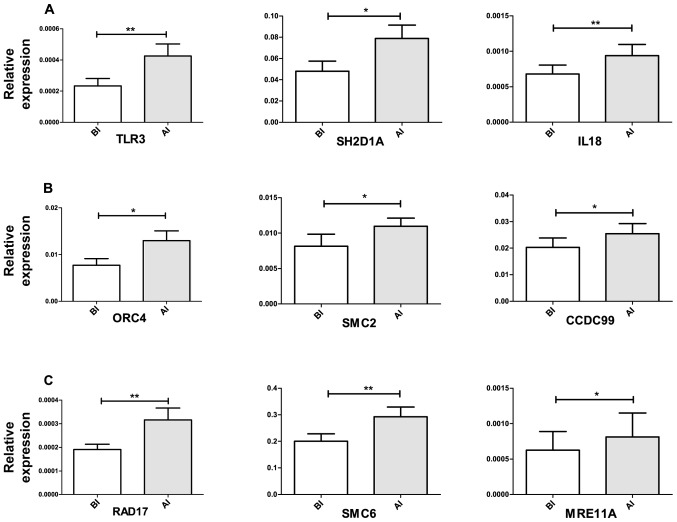
Comparative quantitative RT-PCR validation on genes differentially expressed. (A) Pro-inflammatory response stimulation (*TLR3, SH2D1A* and *IL18*); (B) cell cycle progression (*ORC4, SMC2* and *CCDC99*); (C) DNA damage and repair (*RAD17, SMC6* and *MRE11A*). Relative expression levels were calculated using Pffafl method normalized to *PGK1* gene levels. Statistical comparison on the level of induction between the control and irradiated samples was done by applying paired t-test. A p<0.05 was considered as significant difference between the two conditions. ^*^p<0.05, ^**^p<0.005, ^***^p<0.0001. BI, before irradiation; AI, after irradiation.

Similarly, GSEA and EGAN showed downregulation of gene sets involved in fibroblast growth factor signaling. These nodes showed a connection with the adaptive immune response gene set and immune-related network, respectively (Figs. 1 and 5). Downstream immune-related nodes were connected to the positive cell cycle progression and survival via the induction of the gene sets involved in CDC20, ORC1 and CDH1 degradation and promotion of DNA replication (Figs. 1 and 6). Among the upregulated genes contributing to the positive regulation of cell cycle progression are *CCDC99, ORC4* and *SMC2* (Figs. 4 and 7B).

### Low doses of ionizing radiation induces increased DNA damage

For all patients an increase of the γ-H2AX foci yield was observed: 0.47±0.19 foci/cell (Table III). Furthermore, after 18–24 h, significantly upregulated enriched gene sets were determined, and differently expressed genes that are linked to DNA damage and repair signaling like RAD17, MRE11A, and SMC6 were found (Figs. 3 and 7C).

## Discussion

We investigated *in vivo* the biological responses to low doses of ionizing radiation. To this end, we assessed DNA damage, through scoring of γ-H2AX foci, and performed whole genome analysis followed by qRT-PCR validation (Fig. 7) on whole blood samples collected from prostate cancer patients undergoing IMRT. Whole blood samples were collected from prostate cancer patients before, and at 30 min (for γ-H2AX studies), and 18–24 h (for microarray studies) after the first fraction of irradiation. We chose to perform the experiments on whole blood samples as these are composed of a complex combination of different cell types; therefore, it allows the study of a collective tissue response. On the other hand, blood is a circulating tissue, thus it reflects the response to the calculated equivalent total body dose.

### Prostate cancer patients show induction of pro-inflammatory response via the activation of viral signaling

Previously, we demonstrated that low doses of ionizing radiation induce a unique gene expression profile compared to high doses (11). The low doses are characterized by the induction of stimulatory immune response through the activation of chemokine and cytokine signaling, while high doses are characterized by a damaging response through p53 signaling. In agreement with these results, current GSEA showed the enrichment of several immune signaling pathways; top ranked gene sets were related to viral signaling, in specific human immunodeficiency virus (HIV) infection signaling and interferon secretion (Table I). Viral response is composed of signaling network between NF-κB, ERK 1/2 MAP kinase and p38 MAP kinase pathways. Furthermore, it is known that ionizing radiation is able to activate HIV promoter and gene expression in T cells. The gene expression of HIV viral infections are regulated by various cell signaling events that combine mitogens, cytokines, stress, and DNA damage (18). In other words, the enrichment of the HIV-infection and interferon gene sets in our data suggests a ‘communication network’ between DNA damage and central pathways in the immune response (Figs. 1 and 3). In addition, to that, other viral-related gene sets were also shown to be upregulated; these are NEP/SEP viral proteins, subset of the HIV-infection gene set, and degradation of APOBEC3G via VIF (viral infectivity factor). APOBEC3G is a protein that plays a role in activating an antiviral response; its degradation denotes an amplification of viral and inflammatory response (19). One of the key genes that plays a role in response to viral infections is the toll-like receptor 3 (TLR3) (Fig. 7A), after viral infection TLR3 recognizes double strand RNA (dsRNA) that leads to downstream activation of type I interferons and NF-κB, a proinflammatory and prosurvival pathway (20,21). TLR3 was reported also to be activated upon interaction with exogenous and endogenous RNA molecules (22). Furthermore, GSEA showed the enrichment of TLR cascade gene set (Table I), where TLR3, 7 and 8, involved in viral signaling, contributed to the enrichment score (23). In addition, several genes playing a role in viral signaling and interferon induction such as *IFIH1, MAPK8, KPNA4* and *IL18* genes were shown to be upregulated (Fig. 3).

Downstream of the activation of TLRs and interferons is the NF-κB signaling pathway, where EGAN analysis showed deregulation of IκB proteins (Table II). Overexpression of *SH2D1A* and *IL18* (Fig. 7A), and *CUL1* may indicate the positive regulation of NF-κB signaling (Fig. 3) (24–26).

### Prostate cancer patients show induction of pro-inflammatory response via the activation of adaptive and innate immune signaling

Previously, we have demonstrated that low doses induce the activation of T- and B-cell receptors and innate-related gene set, such as toll-like receptors, NOD-like receptors and RIG-like receptors (11). In agreement with these results, GSEA showed the enrichment of several gene sets that are involved in the stimulation of the immune response via the activation of both adaptive and innate immune responses. The second ranked immune gene set was CD28 stimulation, which is related also to the CTLA4 inhibition gene set (Table I). T cell activation is dependent on the opposing signaling from two cell receptors CD28 and CTL4A. Liu and colleagues (27) have reported that stimulation of CD28 is dose-dependent and specific to low doses of ionizing radiation. Furthermore, the same group reported upregulation in CD28 and downregulation of CTLA4 in lymphocytes isolated from mouse blood exposed to 0.075 Gy whole body irradiation. They showed also that the interaction between antigen presenting cells and T cells is suppressed after exposure of mice to 2-Gy whole body irradiation as a result of CTLA4 upregulation (28). In addition, programmed death 1 (PD1) signaling was shown to be upregulated; PD1 is a surface membrane protein that plays a role in attenuating autoimmune responses, thus it acts in response to the increased activity of the T cell signaling (29). Other gene sets related to innate immune response and inflammation were shown to be activated as well; these include phagosome pathway and inflammasome formation.

### Prostate cancer patients show induction of pro-survival response via immune-stimulation and cell cycle progression responses downstream the growth factor signaling

There is growing evidence that low doses of ionizing radiation have a proliferative and pro-survival responses through the involvement of growth factors (11,30–32). Our data showed downregulation in several growth factors pathways, e.g. FGF, IGF-I and PDGF and several molecules involved in extracellular matrix (ECM) molecules (e.g. *LAMB3, COL20A1* and *COL9A3*) that are involved in growth signaling. This could be related to the late time-point. GSEA and EGAN analysis showed that the growth factor signaling cluster showed a connection with the adaptive immune response node and the viral response node; growth factors, such as FGF were previously shown to be involved in an immune-stimulatory reaction in response to lipopolysaccharide (LPS) stimulation (33,34). Furthermore, genes playing a role in the positive regulation of ERK, MAPK and NF-κB signaling were shown to be upregulated, such as the induction of *SOS1, ITGAV, AKT3, PIK3C2A, MAPK8, SH2D1A* and *IL18* (Figs. 3 and 5).

Furthermore, both analyses showed that viral and immune response gene sets were connected to the nodes of the cell cycle progression and DNA replication (Figs. 1, 6 and 7C). Previously, it was reported that regulation of cell cycle is a characteristic of a low dose response 24 h post-irradiation (9,10). In contrast to our expectation, cell cycle was not arrested and cell cycle checkpoints were not activated, probably due to the low doses received by the patients and the cell cycle positive regulation of the downstream growth factor and immune stimulation.

### Prostate cancer patients show increased DNA damage and anti-apoptotic response post-IMRT

DNA damage signaling was induced 30 min post-irradiation (Table III) and did not terminate 18–24 h later (Figs. 3 and 7B). Taking into account that the cell cycle arrest was not activated (Fig. 4), and was shown not to be launched under a threshold of 200 mGy; this might increase the possibility of carrying unrepaired or misrepaired DNA breaks through the cell division process, thus induction of cancers would be more probable (35,36). In addition, p53 signaling, which is known to be a central player in response to ionizing radiation (37), was not enriched in either analysis approach. The DNA damage and repair response was accompanied by an anti-apoptotic response; where genes involved in stabilization of p53 were downregulated (*PHLDA3*) (38) while others involved in its degradation were upregulated (*MTBP*) (39). *BBC3*, belongs to the BH3-only pro-apoptotic genes, and was also downregulated.

In conclusion, our study demonstrated that immune-stimulatory signaling played a central role in response to low doses of ionizing radiation. These results are in agreement with those reported previously in our *in vitro* whole genome analysis (11). Furthermore, we showed that responses to low doses are a communication network between growth factors and cell cycle progression pathways stimulated by immune signaling. Moreover, we report that remaining unrepaired DNA damage still exists after 18–24 h.

Our study addresses the need for reconsideration of the health risks from the out-of-field low doses of ionizing radiation exposed to the normal tissues when undergoing IMRT. Inflammatory and DNA damage responses may carry the risk of development of systematic inflammations and secondary cancers, respectively; there is accumulating number of studies that show advantages of using particle therapy over treatments that use X-rays. It is demonstrated that the healthy surrounding tissues are spared from out-field radiation. However, other studies have reported that secondary neutrons can carry the risk of developing secondary cancers during particle therapy. There are still no clear-cut answers for a ‘perfect’ radiotherapy approach, and further inter-disciplinary research is still required (7,40).

## Figures and Tables

**Table I.** Statistical significance of GSEA Reactome database gene sets.

Category	Gene set	Size of gene set	FDR q-value
Immune signaling	HIV^a^ infection	181	<0.0001
	Interferon secretion	63	<0.0001
	NEP^b^/SEP2^c^ viral proteins	25	<0.0001
	Activation of APOE3G^d^ degradation via VIF^e^	47	0.001
	CD28^f^ stimulation	56	0.004
	Antigen processing and presentation	183	0.006
	BCR^g^ activation	115	0.02
	TCR^h^ activation	13	0.009
	Phagosome pathway	55	0.01
	Adaptive immune response	460	0.013
	Inhibition of CTLA4^i^	20	0.013
	Inflammasomes	15	0.02
	Activation of NFκB	60	0.031
	Activation of TLR^j^ signaling	11	0.03
	Cytokine signaling	237	0.041
	NLR^k^ signaling	38	0.042
	IL7 signaling	10	0.045
	PD1^l^ signaling	15	0.047
Cell cycle	Mitotic cell cycle	278	<0.0001
	Degradation of mitotic proteins via CDC20^m^	61	<0.0001
	Degradation of CDH1^n^	54	<0.0001
	Removal of CDC6^o^	45	<0.0001
	DNA replication	170	<0.0001
	Chromosome maintenance	100	0.028
	ORC1^p^ removal	58	0.003
DNA damage and repair	DNA repair	91	<0.0001
	Formation of NER^q^ complex	17	0.0009
	Fanconi anemia	16	0.001
Growth signaling	FGFR^r^ activation	21	<0.0001
	SHC^s^ cascade	25	0.001
	PI3K^t^ cascade	51	0.029
	IGFBPs^u^	14	0.023
Metabolism	Amino acids metabolism	16	<0.0001
	Metabolism of lipids	19	0.001
	Metabolism of proteins	24	0.002
	TCA^v^ cycle	105	0.006
	Glucose transport	36	0.031

a HIV, human immunodeficiency virus;

b NEP, nuclear export protein;

c SEP2, septin-2;

d APOBEC3G, apolipoprotein B mRNA-editing, enzyme-catalytic, polypeptide-like 3G;

e VIF, viral infectivity factor;

f CD28, custer of differentiation 28;

g BCR, B-cell receptor;

h TCR, T-cell receptor;

i CTLA4, cytotoxic T-lymphocyte antigen 4;

j TLR, toll-like receptor;

k NLR, NOD-like-receptor;

l PD1, programmed death 1;

m CDC20, cell divion cycle protein 20;

n CDH1, cadherin-1;

o CDC6, cell divion control protein 6 homolog;

p ORC1, origin recognition complex subunit 1;

q NER, nucleotide excision repair;

r FGFR, fibroblast growth factor receptor;

s SHC, Src homomogy 2 domain containing transforming protein;

t PI3K, phosphatidylinositol 3- and 4-kinase;

u IGFBP, insulin growth factor binding proteins;

v TCA, tricarboxylic acid cycle.

**Table II.** Enriched pathways of the differentially expressed genes.

Pathway	p-value
Growth factors signaling and cell cycle progression	
Extracellular matrix proteins	1.16E-19
Extracellular signal regulated MAP kinases	2.90E-16
Mitotic cell cycle	8.70E-16
Insulin growth factor I	2.30E-15
Fibroblast growth factors	3.45E-11
DNA replication	1.98E-10
Signaling by platelet derived growth factor	2.80E-04
Viral and immune response	
Virus replication	2.80E-06
DNA damage and repair	4.90E-06
Toll-like receptors	5.40E-06
IκB proteins	1.90E-05
Metabolism	
Metabolism of lipids and lipoproteins	1.40E-05
Metabolism of proteins	5.40E-04
RNA degradation	6.40E-04

**Table III.** Number of induced γ-H2AX foci and the equivalent total body dose (ETBD) in the eight prostate cancer patients 30 min post-IMRT.

	Induced foci/cell	ETBD (mGy)
Patient 1	0.622	28.01
Patient 2	0.584	46.34
Patient 3	0.267	30.24
Patient 4	0.674	33.79
Patient 5	0.583	37.94
Patient 6	0.561	25.54
Patient 7	0.28	22.07
Patient 8	0.194	23.86
